# Diffusion-weighted imaging for determining response to neoadjuvant therapy in pancreatic cancer: a systematic review and meta-analysis

**DOI:** 10.1007/s00330-023-10381-0

**Published:** 2023-11-01

**Authors:** Carlos Bilreiro, Luísa Andrade, Rui Mateus Marques, Celso Matos

**Affiliations:** 1 Radiology Department, Champalimaud Foundation, Lisbon, Portugal; 2 Champalimaud Research, Champalimaud Foundation, Lisbon, Portugal; 3 Nova Medical School, Lisbon, Portugal; 4 Radiology Department, Hospital de S. José, Lisbon, Portugal

**Keywords:** Diffusion magnetic resonance imaging, Neoadjuvant therapy, Pancreatic carcinoma, Meta-analysis

## Abstract

**Objectives:**

To determine the role of diffusion-weighted imaging (DWI) for predicting response to neoadjuvant therapy (NAT) in pancreatic cancer.

**Materials and methods:**

MEDLINE, EMBASE, and Cochrane Library databases were searched for studies evaluating the performance of apparent diffusion coefficient (ADC) to assess response to NAT. Data extracted included ADC pre- and post-NAT, for predicting response as defined by imaging, histopathology, or clinical reference standards. ADC values were compared with standardized mean differences. Risk of bias was assessed using the Quality Assessment of Diagnostic Studies (QUADAS-2).

**Results:**

Of 337 studies, 7 were included in the analysis (161 patients). ADC values reported for the pre- and post-NAT assessments overlapped between responders and non-responders. One study reported inability of ADC increase after NAT for distinguishing responders and non-responders. A correlation with histopathological response was reported for pre- and post-NAT ADC in 4 studies. DWI’s diagnostic performance was reported to be high in three studies, with a 91.6–100% sensitivity and 62.5–94.7% specificity. Finally, heterogeneity and high risk of bias were identified across studies, affecting the domains of patient selection, index test, reference standard, and flow and timing.

**Conclusion:**

DWI might be useful for determining response to NAT in pancreatic cancer. However, there are still too few studies on this matter, which are also heterogeneous and at high risk for bias. Further studies with standardized procedures for data acquisition and accurate reference standards are needed.

**Clinical relevance statement:**

Diffusion-weighted MRI might be useful for assessing response to neoadjuvant therapy in pancreatic cancer. However, further studies with robust data are needed to provide specific recommendations for clinical practice.

**Key Points:**

*•The role of DWI with ADC measurements for assessing response to neoadjuvant therapy in pancreatic cancer is still unclear.*

*•Pre- and post-neoadjuvant therapy ADC values overlap between responders and non-responders.*

*•DWI has a reported high diagnostic performance for determining response when using histopathological or clinical reference standards; however, studies are still few and at high risk for bias.*

## Introduction

Pancreatic cancer is one of the most lethal cancers worldwide, with surgical resection as the only potentially curative therapeutic option [1, 2]. Nevertheless, most patients are not surgical candidates, many presenting with locally advanced disease. These patients can undergo neoadjuvant chemotherapy or chemoradiotherapy, attempting to downstage the disease and allowing a successful surgical resection [3]. Evaluating these patients, however, is a difficult task, as imaging studies do not accurately reflect response to neoadjuvant therapy (NAT) [4–7]. Traditional size criteria in cross-sectional imaging are known to be unreliable, as the primary pancreatic lesion may undergo minimal or no size reduction, due to remaining tumoral fibrotic stroma even when a response in the cancer cells has already occurred [8].

Diffusion-weighted MRI (DWI), quantified by apparent diffusion coefficient (ADC), is a cornerstone in the MRI evaluation of pancreatic cancer, especially for diagnostic purposes [9]. This technique probes microstructural characteristics of biological tissues, namely cell density and extracellular compartment’s composition, therefore being regarded as a useful biomarker for characterizing neoplastic lesions [10, 11]. However, its value in the assessment of response to NAT in pancreatic cancer is still undetermined, as studies have reported inconclusive and diverse results, both regarding the classification of patients as responders and non-responders and the correlation with histopathological response [12–16].

This study aims to determine the role of DWI in the evaluation of response to NAT in pancreatic cancer, by systematically reviewing the published literature and performing a meta-analysis of reported data.

## Materials and methods

This study is presented according to the Preferred Reporting Items for Systematic Reviews and Meta-Analyses (PRISMA) [17]. The study protocol was registered in an international database prior to data search and collection — PROSPERO: CRD42022309467 [18]. Eligibility criteria were defined according to the following PICOs: (P) patients with pancreatic cancer undergoing neoadjuvant chemotherapy, radiotherapy, or chemoradiotherapy; (I) diffusion-weighted MRI (DWI) is the index test; (C) no comparison is to be performed; (O) outcome is assessed with an acceptable reference standard for determining response status to NAT — post-operative histopathology, clinical follow-up, imaging follow-up.

### Search strategy

MEDLINE, EMBASE, and Cochrane Library (Cochrane Database of Systematic Reviews, Cochrane Central Register of Controlled Trials, Cochrane Clinical Answers) databases were used for the literature search, and the final search was undertaken on 17/01/2023. The search strategy was first defined by an abdominal radiologist with 4 years of experience (C.B.), and reviewed by two radiologists (L.A. and C.M., with 7 and 35 years of experience, respectively). The search query including title and abstract was the following: “pancreatic OR pancreas AND neoadjuvant OR chemotherapy OR radiotherapy OR chemoradiotherapy AND diffusion OR apparent diffusion coefficient OR ADC OR diffusion-weighted MRI OR DWI.” Only studies in the English literature were included; study publication dates were not restricted to a certain period.

The search results were first filtered for relevance by title and abstract review by two reviewers (C.B. and L.A.); the same reviewers then independently performed the full-text reviews of the resulting studies, determining if these fulfilled the criteria for inclusion and solving discrepancies in a consensus meeting. Finally, reference lists of the included studies were additionally searched for more relevant studies.

### Inclusion criteria

Patients were included according to the following: diagnosis of pancreatic cancer; NAT was performed with chemo-, radio-, or chemoradiotherapy; DWI with retrievable ADC measurements was reported for tumor imaging before, after, or before and after NAT; a measure of response to NAT was provided, including histopathological response scores, clinical follow-up evaluation, correlation with RECIST criteria.

### Exclusion criteria

If studies were found using overlapping groups of patients, one of the studies would be excluded to avoid duplication of included patients. Studies where a reference standard was not used to determine response to NAT were excluded. Studies including metastatic (stage IV) patients were excluded, in order to exclude patients undergoing palliative and not neoadjuvant therapy. As only human data was collected, studies performed in animals were excluded. Conference abstracts, letters, and comments were excluded from the analysis.

### Risk of bias assessment

A QUADAS-2 assessment tool was used, based on the domains of patient selection, index test, reference standard, and flow and timing [19]. The tool was adapted to the current review by C.B. and L.A. in consensus, defining quality standards for each of the domains assessed, and then applied by both authors independently to the included studies. Discrepancies in evaluation between both readers were solved in a consensus meeting.

### Data extraction

Two authors (C.B. and L.A.) independently extracted the following data: first author name, year of publication, journal name, type of study (retrospective, prospective), institution(s) and study dates, number of patients, patients’ age, resectability status, chemotherapy and/or radiotherapy details (protocols and timings), MR system field strength, DWI acquisition *b* values, DWI timing (before and/or after NAT), MRI readers’ experience, tumor location in the pancreas, tumor size pre- and post-NAT, ADC values pre- and post-NAT for responders and non-responders, histopathological analysis for NAT response grading, clinical follow-up data when used to determine response to NAT.

Whenever possible, ADC data was retrieved as a 2 × 2 table, as defined: true positives were cases where a positive index text was confirmed with the reference standard; true negatives were cases where a negative index test was confirmed with the reference standard; false positives were cases where a positive index test was not confirmed with the reference standard; false negatives were cases where a negative index test was not confirmed with the reference standard.

When available, Pearson’s correlation coefficient values between ADC and histological response grades were retrieved, including ADC measurement timing (pre- or post-NAT) and histological grading system (based either on Evans or College of American Pathologists classification systems) [20, 21].

When the published data for any specific study was considered insufficient or incomplete, an attempt to contact the study’s corresponding author by e-mail was conducted, and the new data added to the analysis, if then provided.

### Data analysis

The collected data regarding pre-NAT ADC values in responders and non-responders were summarized in forest plots.

Standardized mean differences with random effects and *Z* test for overall effects were used for comparing ADC values in the pre-NAT period, between responders and non-responders.

Sources of heterogeneity between studies were recorded and qualitatively discussed [22].

Data extraction and analysis were performed using Microsoft Excel® and Review Manager (RevMan) 5.4. (The Cochrane Collaboration, 2020).

## Results

**Fig. 1:**
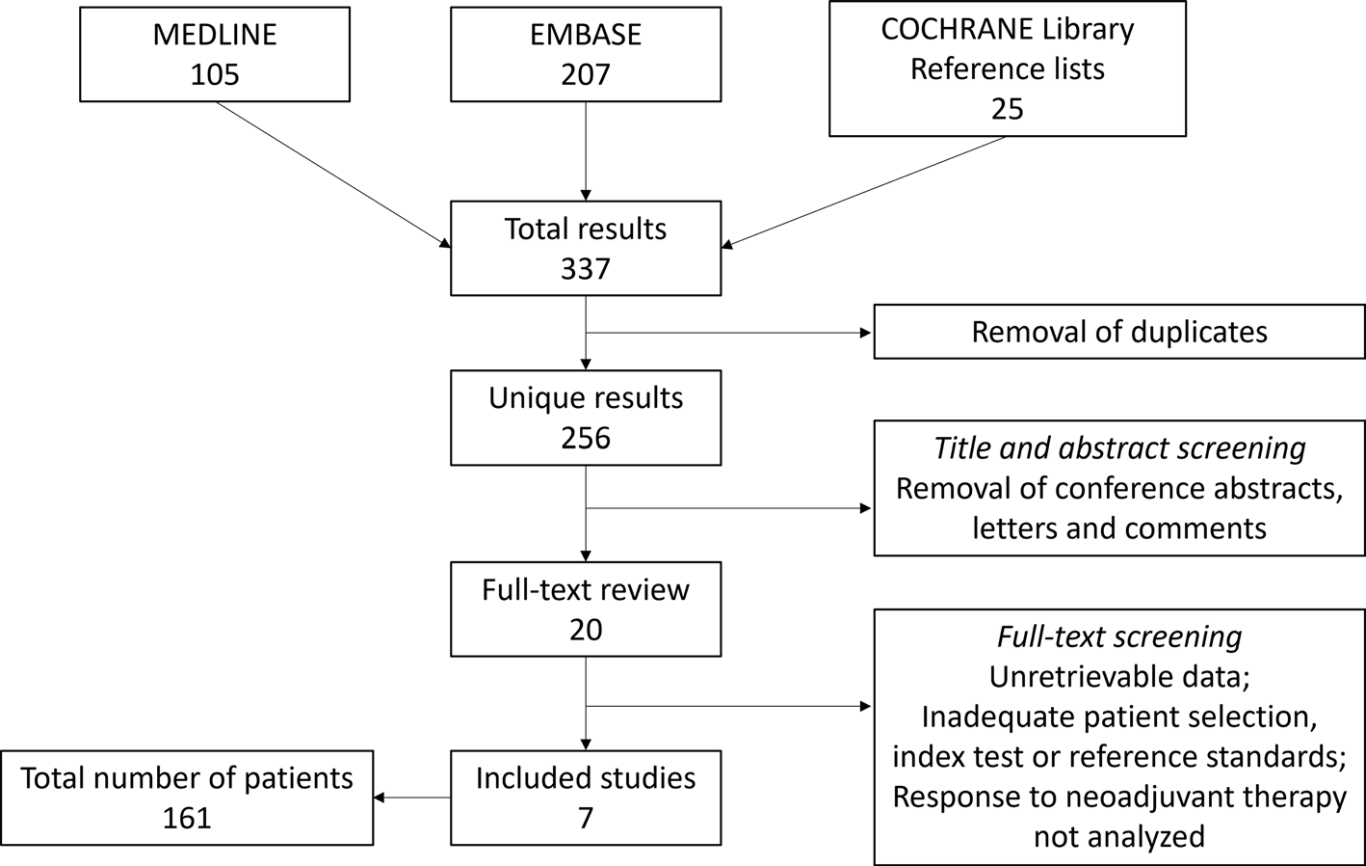
Study flowchart

The initial search yielded 337 studies; after removal of duplicates, title and abstract screening, and full-text screening, 7 studies with 161 patients (6–41 patients per study) were finally included (Fig. 1) [12–14, 23–26]. One study’s corresponding author was contacted with a request for further information: however, a reply was not obtained.

### Studies’ and patients’ characteristics

The included studies’ characteristics are described in Table 1. Most studies were recent (6/7 from 2017 onwards; date range of publication: 2014–2022), and prospective (5/7 prospective). Five out of seven studies were performed on 3-T MRI systems, and most (6/7) used high *b* values for DWI (800 s/mm^2^ and higher). All studies used mono-exponential models for ADC calculations. The number and experience of readers were inconsistently reported. Most studies (5/7) performed DWI before and after NAT. For determining response to NAT, 4 studies used histopathological criteria (3 using a modified Evans classification, 1 using the College of American Pathologists classification), 2 studies used RECIST 1.1 criteria, and 1 study used clinical criteria (multidisciplinary assessment of surgical indication).

**Table 1:** Studies characteristics

Study	Journal	Year	Study design	Magnetic field	*B* values (s/mm^2^)	Number of readers	Readers experience	ROI drawing process	DWI timing	Reference standard
Cuneo KC, et al [23]	*Transl Oncol*	2014	Prospective	ND	0, 100, 500, 800	2	Radiologist + researcher (ND)	Manual delineation of tumor volume	Before NT	Pathological (Evans)
Dalah E, et al [24]	*Transl Oncol*	2018	Retrospective	3 T	0, 500, 1000	ND	ND	Manual delineation of tumor volume	After NT	Pathological (CAP)
Hussien N, et al [12]	*Front Oncol*	2022	Prospective	3 T	0, 400, 800	ND	ND	ND	Before and after NT	Clinical (multidisciplinary — surgical indication)
Kang JH, et al [14]	*Eur Radiol*	2021	Prospective	3 T	0, 30, 60, 120, 200, 400, 800	2	Radiologists (8 + 10 years)	Average of two largest manual cross-sectional ROIs; intra- and inter-observer variability assessed	Before and after NT	RECIST 1.1 + tumor-vessel contact
Kinh Do R, et al [25]	*Tomography*	2020	Prospective	3 T	0, 500	ND	Radiologists + radiation oncologists (ND)	Manual delineation of tumor ROIs	Before and after NT	RECIST 1.1
Okada KI, et al [26]	*J Hepatobiliary Pancreat Sci*	2017	Retrospective	1.5 T	0, 50, 800	2	Radiologists (2)	Manual ROI: largest cross-sectional area based on CT	Before and after NT	Pathological (Evans)
Okada KI, et al [13]	*Langenbecks Arch Surg*	2020	Prospective	3 T	0, 50, 1000	1	Radiologist (1)	Manual ROIs: 3–4 largest cross-sectional areas based on CT	Before and after NT	Pathological (Evans and CAP)

*ND*, non-disclosed; *NT*, neoadjuvant therapy; *NM*, nuclear medicine consultant; *CAP*, College of American Pathologists; *CT*, computerized tomography; *ROI*, region of interest

Included patients’ characteristics are summarized in Table 2. The number of patients included in each study ranged from 6 to 41, and the patients’ mean age ranged from 52.5 to 69 years. Most studies (5/7) included borderline resectable patients; 2/7 included locally advanced patients. Six studies described tumor locations, most often in the head of the pancreas (5/6). Mean tumor size pre-NAT ranged from 27.8 to 51.5 mm, and post-NAT ranged from 22.3 to 55.5 mm. Chemotherapy regimens varied, gemcitabine being included in most studies (6/7); 4 studies included radiotherapy.

**Table 2:** Patient, disease, and treatment data collected

Study	Number of patients	Age (years)	Initial staging	Resectability criteria	Tumor size (pre-NT)	Tumor size (post-NT)	Tumor location	Resection surgery	Chemotherapy	Radiotherapy
Cuneo KC, et al [23]	10 (7*)	60.7	Resectable	ND	ND	ND	ND	All patients	Gemcitabine + oxaliplatin	30 Gy in 2 fractions (method ND)
Dalah E, et al [24]	25	ND	Resectable and borderline	ND	37.79 ± 12.1 cc	9.75 ± 5.93 cc	100% head	All patients	Gemcitabine or Capecitabine + FOLFIRINOX or gemcitabine-Abraxane	50.4 Gy in 28 fractions, IMRT
Hussien N, et al [12]	30	52.5	Borderline	NCCN (v2.2012)	ND	ND	23.3% head 76.7% body/tail	11 patients	63.3% FOLFIRINOX 36.7% gemcitabine/cisplatin	No
Kang JH, et al [14]	41	60.3	Borderline and locally advanced	NCCN (v2.2017)	36 ± 8	ND	65.9% head 34.1% body/tail	25 patients	FOLFIRINOX	No
Kinh Do R, et al [25]	10 (6*)	64	Locally advanced	ND	51.5 ± 24.3	R: 42 ± 2.8 NR: 55.5 ± 26	50% head 50% body/tail	None	80% FOLFIRINOX 10% GEM/NAB 10% FOLFIRINOX + GEM/NAB	27–33 Gy in 3 fractions, SBRT
Okada KI, et al (2017) [26]	24	67	Borderline	NCCN (v2.2015)	28.9 ± 12.5	26.9 ± 12.2	54.2% head 45.8% body/tail	All patients	S-1 + gemcitabine or FIRINOX or GEM/NAB or S-1 + radiation	50 Gy, EBRT (4 patients)
Okada KI, et al (2020) [13]	28	69	Borderline	NCCN (v2.2015)	27.8 ± 9.36	22.3 ± 9.8	68% head 32% body/tail	All patients	GEM/NAB	No

^*^Number of patients eligible for response assessment. If not otherwise mentioned, tumor size is expressed in mm. *ND*, non-disclosed; *R*, responders; *NR*, non-responders; *pre-NT*, pre-neoadjuvant therapy; *post-NT*, post-neoadjuvant therapy; *NCCN*, National Comprehensive Cancer Network

### DWI for response assessment

Table 3 summarizes ADC measurements and their performance in assessing response to NAT. ADC values overlapped for responders and non-responders between studies: in the pre-NAT period, these ranged from 1.0 to 1.61 × 10^−3^ mm^2^/s for responders, and from 1.25 to 1.5 × 10^−3^ mm^2^/s for non-responders. In the post-NAT period, only one included study reported ADC values: 1.4 × 10^−3^ mm^2^/s for responders, and 1.3 × 10^−3^ mm^2^/s for non-responders. One study reported ADC increase from the pre- to the post-NAT period: 14.9% for responders and 10.3% for non-responders.

**Table 3:** ADC measurements and response assessment

Study	R: pre-ADC	NR: pre-ADC	R: post-ADC	NR: post-ADC	R: ADC increase	NR: ADC increase	Response assessment	Diagnostic performance	
								**Criteria**	**Sensitivity/specificity**
Cuneo KC, et al [23]	1.61 ± 0.05	1.25 ± 0.16	ND	ND	ND	ND	Pre-ADC correlates with pathological response grade	NA	NA
Dalah E, et al [24]	ND	ND	ND	ND	ND	ND	Post-ADC correlates with pathological response	NA	NA
Hussien N, et al [12]	1.0 (1.0–1.3)	1.4 (1.0–1.4)	1.4 (1.3–1.7)	1.3 (1.0–1.7)	ND	ND	ADC increase in responders	Stationary vs. regressive ADC	100%/94.7%
Kang JH, et al [14]	1.46 ± 0.23	1.40 ± 0.19	ND	ND	14.9 ± 13.8%	10.3 ± 27.6%	No significant differences in ADC	NA	NA
Kinh Do R, et al [25]	1.60 ± 0.17	1.50 ± 0.05	ND	ND	ND	ND	No significant differences in ADC	NA	NA
Okada KI, et al (2017) [26]	ND	ND	ND	ND	ND	ND	Pre- and post-ADC correlate with pathological response grade	Pre-ADC (≥ 1.2)	100%/75%
Okada KI, et al (2020) [13]	ND	ND	ND	ND	ND	ND	Pre-ADC, post-ADC, and ADC increase correlate with pathological response grade	Pre-ADC (≥ 1.2)	91.6%/62.5%
								Post-ADC (≥ 1.4)	100%/81%

*R*, responders; *NR*, non-responders; *pre-ADC*, ADC value pre-neoadjuvant therapy; *post-ADC*, ADC value post-neoadjuvant therapy; *ND*, non-disclosed; *NA*, not assessed. ADC values (× 10^−3^ mm^2^/s) are expressed as means ± standard deviations or medians and ranges (in parentheses)

Two studies, both using RECIST 1.1 criteria for assessing response status, reported no significant differences in ADC values for differentiating responders from non-responders [14, 25]. All other studies (5/7) reported ADC being able to determine response status, using histopathological or clinical criteria as reference standard. The 4 studies evaluating histopathological response (Table 4) reported a correlation between both pre- and post-NAT ADC values and response grade, which was positive when using the modified Evan’s classification and negative when using the College of American Pathologists’ classification [13, 23, 24, 26].

**Table 4:** Correlation coefficients of studies using histopathological criteria to determine response to neoadjuvant therapy

Study	Classification	DWI timing	Correlation coefficient
Cuneo KC, et al [23]	Evans*	Pre	0.94* (*p* = 0.001)
Dalah E, et al [24]	College of American Pathologists	Post	− 0.52 (*p* < 0.05)
Okada KI, et al (2017) [26]	Evans*	Pre	0.63 (*p* = 0.001)
		Post	0.41 (*p* = 0.144)
Okada KI, et al (2020) [13]	Evans*	Pre	0.63 (*p* < 0.001)
		Post	0.71 (*p* < 0.001)
		Pre/Post Difference	0.29 (*p* = 0.138)

^*^Modified version of the Evans classification [20]. *Pre*, pre-neoadjuvant therapy; *Post*, post-neoadjuvant therapy

Three studies reported DWI’s diagnostic performance, with sensitivity values ranging from 91.6 to 100% and specificity values from 62.5 to 94.7% [12, 13, 26]. Noteworthy, these studies used different criteria for determining response status: stationary vs. regressive ADC; pre-NAT ADC ≥ 1.2 × 10^−3^ mm^2^/s; post-NAT ADC ≥ 1.4 × 10^−3^ mm^2^/s.

### Synthesis of collected data

**Fig. 2:**
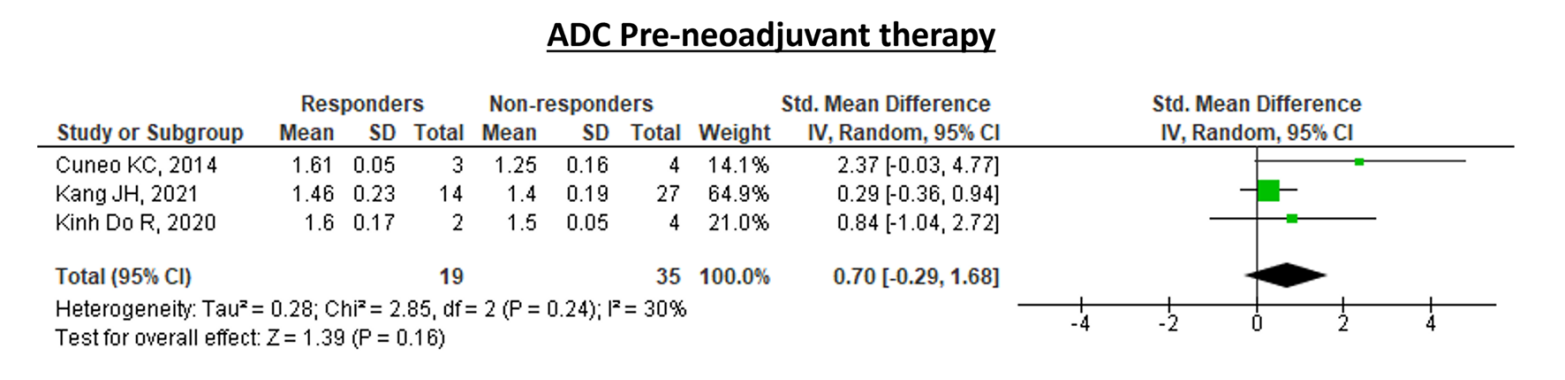
Pooled analysis results of ADC values pre-neoadjuvant therapy. Test for overall effects was not statistically significant, with *Z* = 1.39 (*p* = 0.16)

Figure 2 summarizes the pooled analysis for pre-NAT ADC values reported in responders and non-responders. When testing for overall effects, ADC values were not significantly different between groups of patients (*Z* = 1.39, *p* = 0.16, *I*^2^ = 30%).

Studies were considered too few and heterogeneous to perform a pooled analysis regarding post-NAT ADC values (1 study), ADC variance from pre- to post-NAT (1 study), and diagnostic performance with summary ROC statistics (3 studies with high heterogeneity).

### Risk of bias and heterogeneity

**Fig. 3:**
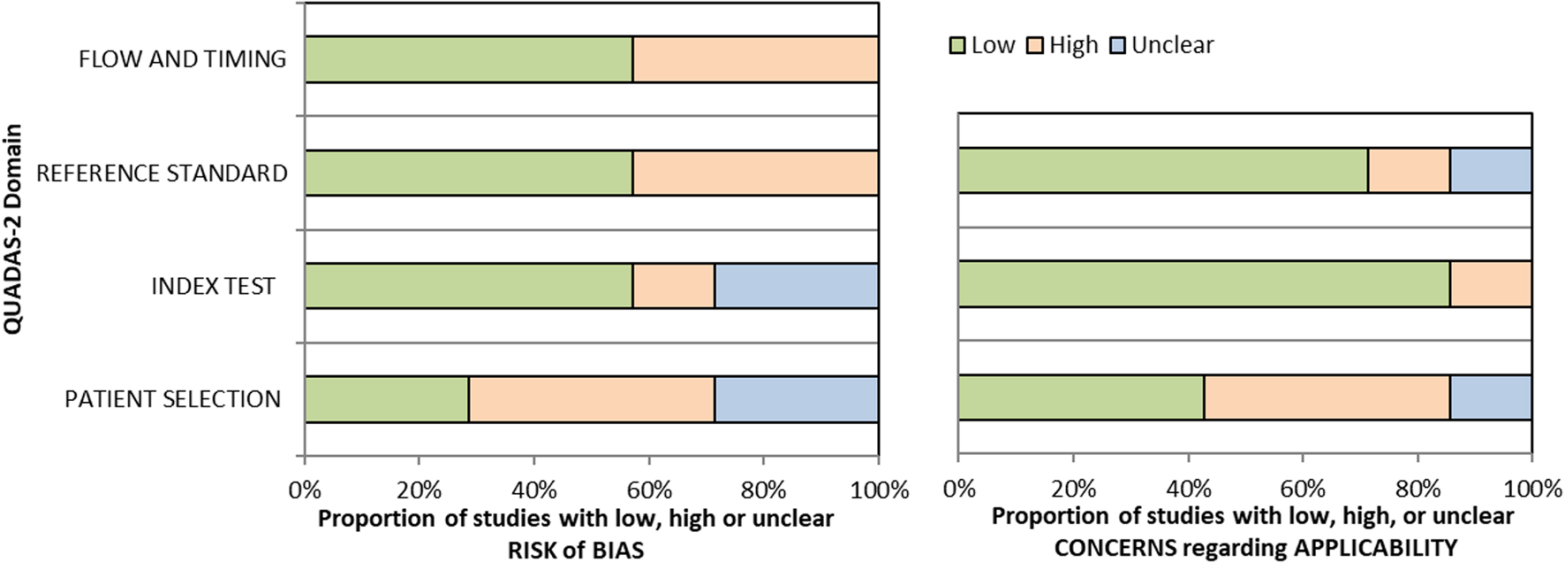
QUADAS-2 chart, representing the estimated risk for bias and applicability concerns for all studies included

Figure 3 illustrates the QUADAS-2 evaluation results, regarding risk of bias and applicability concerns. High risk of bias and applicability concerns were identified across studies, especially for patient selection, but also for the other domains. The main causes for concern regarding patient selection were the exclusion of borderline or locally advanced stages, retrospective studies, non-disclosure of resectability criteria, and patients excluded due to missing or inadequate index test. For the reference standard, the dependance on RECIST criteria, interpretation of the reference standard with knowledge of the index test (DWI), and the use of clinical multidisciplinary evaluation were considered the major causes of concern. Regarding the index test, the major sources of concern stemmed from reader’s number and experience not being disclosed, region of interest (ROI) drawing process not being disclosed or vaguely described, the use of poorly defined diagnostic criteria, and the use of highest *b* values lower than the usual standards (500 s/mm^2^). Finally, in the flow and timing domain, concerns were expressed due to widely variable MRI timings both before and after NAT.

Heterogeneity was also analyzed in a qualitative manner across studies. Regarding the reference standard, the use of RECIST 1.1 criteria was considered a probable source of heterogeneity, as the two studies using it did not report ADC to be of diagnostic value while all others did, when using either clinical or histopathological criteria. For the studies using histopathological response assessment, the correlation coefficients with ADC values were positive when using the modified Evans classification and negative when using the College of American Pathologists classification [20, 21]. Since these classifications’ gradings are the inverse of each other — higher tumor cell destruction translates into higher degrees in the Evans but lower degrees in the College of American Pathologists classifications — the results between studies can be considered concordant, as higher pre- and post-NAT ADC values correlate with higher tumor cell destruction. Another identified source of heterogeneity directly influencing ADC values obtained in each study was the variability in *b* values used for ADC calculation. Although 1/7 studies used low *b* values for ADC calculation, also used for IVIM (intravascular incoherent motion) assessment, this study also used a mono-exponential calculation method for ADC. Also, 1/7 studies used highest *b* values lower than the usual standards (500 s/mm^2^), while all other studies used *b* values of 800–1000 s/mm^2^.

## Discussion

This systematic review determines that DWI with ADC values for the assessment of response to NAT in pancreatic cancer may be useful, with reported high diagnostic performance. However, the included number of studies was small and high risk for bias was identified across studies, prompting a cautious interpretation of these results.

As ADC values observed across studies overlapped for responders and non-responders and no overall effects were seen in the pre-NAT period, an evaluation of absolute ADC values for this purpose appears to not be useful. The reproducibility of ADC values in the pancreas has been shown to be imperfect, and some variability should be expected across individuals, MRI scanners, and even anatomical regions of the pancreas [27–31]. Despite this, a correlation between ADC values and histopathological response was reported in 4 studies, both for the pre- and post-NAT assessments; therefore, high ADC values may still have a role in predicting response to NAT, but more studies providing further characterization in this regard are needed.

Technical issues affecting the reproducibility of ADC values were an important concern in this study, with values varying widely between studies, for both responders and non-responders. For future studies, the use of standardized protocols for image acquisition with motion-robust techniques, post-processing and corrective methods should be effective for diminishing these differences and improve ADC values’ reproducibility [32–37]. Also, the process of ROI placement for ADC measurement, which was inconsistently reported in studies in this review, is known to have inherent variability [38, 39]. This is another aspect that could be improved in further studies, exploring the variability of ADC measurements derived from the measurement process itself, and its effect on the magnitude of ADC differences between groups of responders and non-responders.

The diagnostic performance of DWI for determining response to NAT was high in all three included studies, which is encouraging for implementing its use in clinical practice. Nevertheless, studies used both different DWI-based metrics (1/3 used an ADC increase measure; 2/3 used cutoff ADC values) and different reference standards for this purpose (1/3 clinical; 2/3 histopathological). Therefore, the most adequate methodology when using DWI to differentiate responders from non-responders remains undetermined.

Response assessment after NAT in pancreatic cancer remains a challenge in clinical practice, with most centers relying on both analytical (serum CA19-9 concentration) and imaging data to assess response, the latter based on traditional size criteria: increase in tumor size being suggestive of progressive disease, stability or reduction in tumor size being considered a response and warranting surgical exploration [4, 40, 41]. This approach, however, may lead to unnecessary surgery being performed in cases of persistent advanced disease, or potentially resectable patients being excluded from surgery. Novel biomarkers have therefore been sought, with DWI emerging as a potential imaging candidate, but with inconclusive/insufficient results thus far [42–44]. Our analysis helps establishing DWI with ADC measurement as useful biomarker for assessing response, but current data is still not enough to recommend its implementation in clinical practice.

This systematic review and meta-analysis is limited by the small number of studies, which precluded subgroup analysis and statistical exploration of heterogeneity factors. Studies were also considered heterogeneous and at high risk for bias. We identified similar important sources for both heterogeneity and risk for bias, the most obvious being the variability in resectability status of patients included in each study, NAT regimens, DWI *b* values, readers’ experience, and reference standards (RECIST, clinical and histopathological). In an ideal world, we would expect studies to replicate the best clinical practice and control for interfering variables — include all patients undergoing NAT, use only standardized NAT regimens and DWI acquisition parameters with experienced readers, and use the most accurate reference standards. However, these studies provide real-world data, reflecting the difficulties in managing patients with pancreatic cancer, with imperfect staging systems after NAT, and the evolution of NAT regimens and treatment recommendations in recent years [45, 46].

In conclusion, this systematic review and meta-analysis helps establish DWI as a useful biomarker for determining response to NAT in pancreatic cancer. However, few studies were included and were considered heterogeneous and at high risk for bias. Further data, best provided by studies with standardized procedures for data acquisition and accurate reference standards, are needed.

## Abbreviations

ADC: Apparent diffusion coefficient
DWI: Diffusion-weighted imaging
MRI: Magnetic resonance imaging
NAT: Neoadjuvant therapy
NCCN: National Comprehensive Cancer Network
ROC: Receiver operating characteristics
ROI: Region of interest

## Declarations

### Guarantor

The scientific guarantor of this publication is Dr. Celso Matos.

### Statistics and biometry

One of the authors has significant statistical expertise.

### Study subjects or cohorts overlap

Since this study is a systematic review and meta-analysis, all data was collected from previously published studies.

### Methodology

•diagnostic or prognostic study

•multicentre study
